# Supplementary material for: Non-Coding RNAs Participate in the Regulation of CRY-DASH in the Growth and Early Development of Saccharina japonica (Laminariales, Phaeophyceae)
Source: Int J Mol Sci. 2020 Jan 2;21(1):309. doi: 10.3390/ijms21010309 (PMC6981881; doi:10.3390/ijms21010309)
Supplement: Supplementary file 1 [file ijms-21-00309-s001.pdf]

Supplemenatary Materials

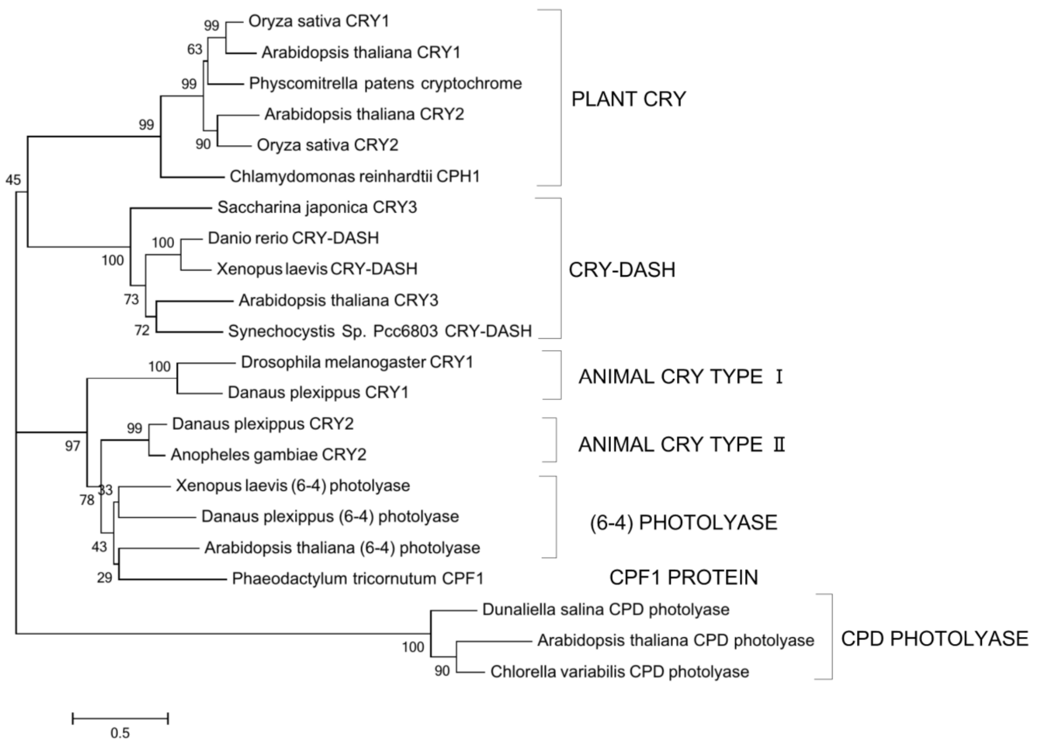

Figure S1. Phylogenetic analysis of CRY-DASHs. In total, 22 protein sequences were used to perform phylogeny analysis using the MEGA 6.0 platform with the neighbor-joining method. Bootstraps of 1000 replicates are expressed in percentage.

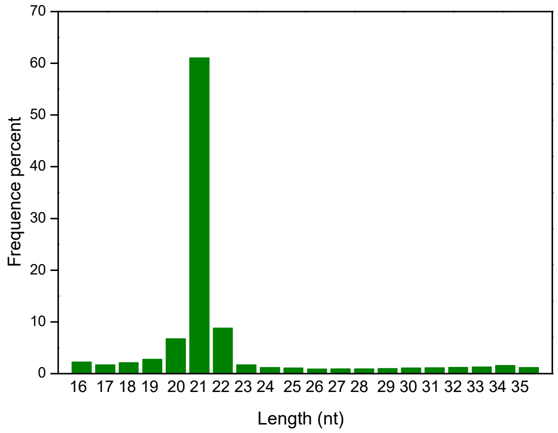

Figure S2. Length distributions of small RNAs in *S. japonica*.

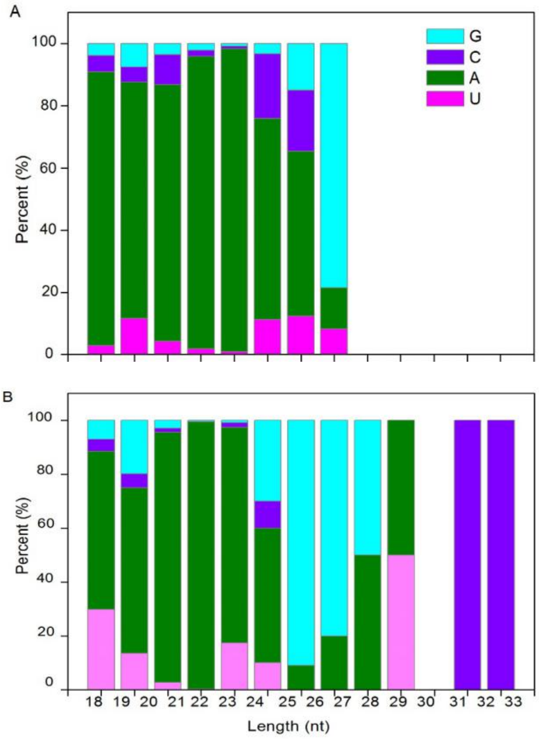

Figure S3. Base bias on the first site of novel (**A**) and known (**B**) miRNAs with specific lengths.

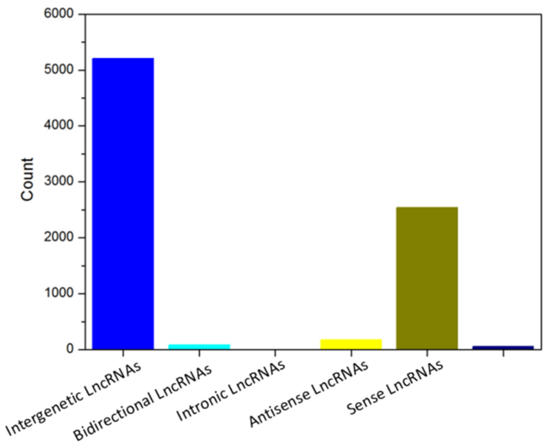

Figure S4. Classification of lncRNA based on their position.

Table S1. Summary of small-RNA seq reads mapping to *S. japonica* reference genome. The suffixes -1, -2 indicate biological replicates for each sample.

Sample	Total Reads	Match Reads	Ratio
sjDR-1	17021748	12137953	71.31%
sjDR-2	14696034	10186706	69.32%
sjBL-1	13404621	9518639	71.01%
sjBL-2	13674165	9969389	72.91%
sjWL-1	13199760	9539465	72.27%
sjWL-2	9352909	6478724	69.27%

Table S2. Summary of lncRNA library reads mapping to the reference genome of *S. japonica* in strand-specific RNA-seq library. The suffixes-1, -2 indicate biological replicates for each sample.

Sample	Total reads	Match reads	Ratio
sjDR-1	86003128	77156097	89.71%
sjDR-2	93901340	80952915	86.21%
sjBL-1	104039374	95201422	91.51%
sjBL-2	86321882	80310174	93.04%
sjWL-1	113955214	104651692	91.84%
sjWL-2	78715228	71700462	91.09%

Table S3. List of primers used in the study.

Primer	Sequence (5′ to 3′)	Description
CRY-DASH-5O	CCCCAAGTGGAATATGGAGTTGCCATTC	5′ RACE
CRY-DASH-5I	CCCGCCGTCTCGCCCCCCTTGAA	5′ RACE
CRY-DASH-3O	CAAGGAGGTCTGGGGCAATG	3′ RACE
CRY-DASH-3I	CTGCCTTCCGACTTGCCGTT	3′ RACE
CRY-DASH-F	CATATGATGGCGAGCTTTTCATCTGA	ORF amplification
CRY-DASH-R	GAATTCCTAGGAAAACTGCCGAAACG	ORF amplification
qCRY-DASH-F	AATGGAGGTCGGGAAGTAC	qRT-PCR for *SjCRY-DASH*
qCRY-DASH-R	CCCCCAAGTGGAATATGG	qRT-PCR for *SjCRY-DASH*
Actin-F	GACGGGTAAGGAAGAACGG	qRT-PCR for *β-actin*
Actin-R	GGGACAACCAAAACAAGGGCAGGAT	qRT-PCR for *β-actin*
U6-F	TCGGGGACATCCGATAAAATTGGAA	qRT-PCR for *U6*
U6-R	GGACCATTT-CTCGATTTATGCGTGTCA	qRT-PCR for *U6*
novel-m3234-5p	CTCAACTGGTGTCGTGGAGTCGGCAATT CAGTTGAGCCGGTAG	RT-primer
novel-m3234-5p-F	GCCGAGTCCAGCCCGGCGTT	qRT-PCR for novel-m3234-5p
novel-m3234-5p-R	CTCAACTGGTGTCGTGGA	qRT-PCR for novel-m3234-5p
TCONS_00001280-F	TAGTGCATTCGGTCCCCCTT	qRT-PCR for TCONS_00001280
TCONS_00001280-R	ACCAAGCCCGTACCAACTCT	qRT-PCR for TCONS_00001280
TCONS_00002718-F	AGCGCACTGCTGTAACTAACTGC	qRT-PCR for TCONS_00002718
TCONS_00002718-R	TCACTTGTTCGCGTCCACGTTG	qRT-PCR for TCONS_00002718
TCONS_00006247-F	AGCTCCACGCACGTTATTCTTCAG	qRT-PCR for TCONS_00006247
TCONS_00006247-R	AATGACACAGCAGTACCACGACAG	qRT-PCR for TCONS_00006247
TCONS_00008286-F	CGTGACGACTCCACATTCCA	qRT-PCR for TCONS_00008286
TCONS_00008286-R	GGCGTGGTAATCGGGTTGAA	qRT-PCR for TCONS_00008286
TCONS_00017519-F	TGCTGCGATGACCTGAACTAACTG	qRT-PCR for TCONS_00017519
TCONS_00017519-R	TGGTGCAGTTGGTGTAACAGGTAC	qRT-PCR for TCONS_00017519
TCONS_00009907-F	GACGACGACGTATGGATTGGAACC	qRT-PCR for TCONS_00009907
TCONS_00009907-R	TAGCTCTGCCGCCGAACATCTAG	qRT-PCR for TCONS_00009907
TCONS_00043396-F	ACGATTACAAGGTGGCACGG	qRT-PCR for TCONS_00043396
TCONS_00043396-R	CTGTTTGCTCGGCTGGCAT	qRT-PCR for TCONS_00043396
TCONS_00043393-F	TCAGAGAGTGACAGCAAGGCGG	qRT-PCR forTCONS_00043393
TCONS_00043393-R	CGGCCCGGGTTGAGCTTTAC	qRT-PCR forTCONS_00043393
TCONS_00008371-F	GGCTGACGCCGCTCGCTA	qRT-PCR forTCONS_00008371
TCONS_00008371-R	GCATCCCGAGCATGTTTTGCA	qRT-PCR forTCONS_00008371

Phylogenetic Analysis

Alignments of cryptochrome and photolyase protein sequences were performed with the ClustalW algorithm using the following parameters: for pairwise alignments, a gap opening penalty of 10 and a gap extension penalty of 0.1. For multiple alignments, a gap opening penalty of 10 and a

gap extension penalty of 0.2 were used. A Gonnet weight matrix was used with residue-specific penalties ON, hydrophilic penalties ON, a gap separation distance of 4, end gap separation OFF, negative matrix OFF, and a delay divergent cutoff of 30%. A phylogenetic tree was constructed by the neighbour-joining method [1], and a bootstrap consensus tree was obtained after 1000 replications. Parameters were set as follows: Poisson correction model, complete deletion of gaps/missing data, and uniform rates among sites.

Reference

1. Saitou N, and Nei M. The neighbor-joining method: A new method for reconstructing phylogenetic trees. *Mol. Biol Evol*. 1987; 4:406-425.
